# Impact of COVID-19 on Outpatient Malnutrition Centers in Urban and Rural Burkina Faso

**DOI:** 10.4269/ajtmh.22-0714

**Published:** 2023-06-12

**Authors:** Paul Ouedraogo, Virginio Pietra, Richard Fabian Schumacher

**Affiliations:** ^1^ Hôpital St Camille de Ouagadougou, Ouagadougou, Burkina Faso;; ^2^ Medicus Mundi Italia, CMA St Camille, Nanoro, Burkina Faso;; ^3^ Medicus Mundi Italia, Brescia, Italy;; ^4^ Ospedale dei Bambini, ASST Spedali Civili, Brescia, Italy

## Abstract

Although the numbers of SARS–CoV-2 infections and related deaths are relatively low in sub-Saharan Africa, the pandemic might lead to a high indirect death toll there. We determined the impact of the COVID-19 pandemic on the management of malnourished children in urban and rural areas. We analyzed data from two Centers for Rehabilitation, Education & Nutrition (CRENs), one in the capital and one in a rural center, both run by the Camillian Fathers. We compared data from the year before the pandemic (2019) with the first 2 years during the pandemic (2020/2021). In the urban CREN, there was a sharp reduction in new patients enrolled, from 340 in the pre-pandemic year to 189 during the first pandemic year and 202 in the second year. The follow-up was significantly shorter during the first pandemic year, with a rebound in the second year (pre: 57 days versus 42 and 63 days for the first and second years, respectively). In the rural CREN, the situation was different: The numbers of patients did not show any significant variation between the pre-pandemic year (191) and the first and second pandemic years (223 and 179, respectively). Different perceptions of the pandemic in urban (high, more testing, more COVID) and rural (low, less information and testing) areas may partly explain this difference. The discrepancy between the decreasing numbers of malnourished children in specialized care during the pandemic—especially in the urban area—is contrary to the lockdown-induced increase in food insecurity and warrants attention to avoid an increase in the silent epidemic of malnourished children in Africa.

## INTRODUCTION

Burkina Faso is a land-locked country in western Africa with 21 million inhabitants. Nearly 13 million face a quantitative and qualitative lack of food. The periodic droughts that characterize the Sahel, leading to crop failure, are further aggravated by continuing terrorist attacks and political instability, leading to more than 2 million internally displaced people. Reduced supply, poor quality, and increased prices have a particular impact on children: More than one-third of those under 5 years old are malnourished.^1^

The Order of the Camillian Fathers has been working on maternal and child health in Burkina Faso for more than 50 years. With the support of Medicus Mundi Italia and the University of Brescia, Italy, they were among the first to introduce zinc supplementation and ready-to-use therapeutic food and later implemented the “first 1,000 days” approach^2^ while participating in the development of dedicated guidelines.^3^ Today, they run two Centers for Rehabilitation, Education & Nutrition (CREN), one based in the Hôpital St Camille de Ouagadougou (HOSCO), located in the capital, and another one in the Center Médical Saint Camille de Nanoro, serving a rural area in the Boulkiemdé region, roughly 100 km to the northwest. In both facilities, trained staff take care of children up to the age of 5 years with acute malnutrition and educate their respective caregivers. Children with good appetite and no medical complications are treated as outpatients, whereas those suffering from complicated severe acute malnutrition or presenting with medical complications are hospitalized. Both centers are well equipped and follow the same management algorithm. They are able to produce on their own therapeutic milk according to the WHO recipe (e.g., the F75 or F100) or peanut-based food (MiSoLa^4^) for the transition phase, in case national suppliers happen to be out of stock. Whenever feasible and available, ready-to-use therapeutic food is also used to save cost and time.^5^ Thus, comparing data from both settings allows us to document the well-known subnational health inequities, which persist especially between the capital and rural communities.^6^

When COVID-19 hit Burkina Faso in March 2020, it was expected that it would be especially problematic because in 2019 there had already been a major food crisis, and a high burden of population-level malnutrition is considered a driver of fatal COVID-19.^7^ Although the numbers of infections and related deaths initially were lower in sub-Saharan Africa than in other world regions for several reasons,^8^ it was feared that the SARS-CoV-2 pandemic might lead indirectly to a catastrophic death toll, as has been observed during and after epidemics^9^ or conflicts^10^ in less-resilient healthcare systems. Travel restrictions, home isolation and the resulting lack of access to school feeding, community lockdowns, and market closures all contribute to aggravation of food insecurity, both in terms of scarce quantity and quality.^11^ Furthermore, micro- and/or macronutrients are not picked up or consultation is delayed for fear of being infected with SARS-CoV-2 from visits to health facilities, where the scarce resources may have been diverted to adult care, putting children’s needs behind. Routine immunizations—comprising rotavirus vaccines—could be interrupted, adding to the possible indirect impact of the pandemic on children and especially the most fragile among them, those who are malnourished.^12^ Wasted children are immunologically compromised and at increased risk of death. In fact, malnutrition is a known contributing factor in almost half of child deaths from both communicable and noncommunicable diseases.^13^ In addition, although acute infections transmitted by person-to-person contact, such as pneumonia and diarrhea, may (at least temporarily) decrease as long as pandemic prevention measures are in place, chronic conditions such as malnutrition are expected to rise. Considering these reasons, a study financed by the Bill & Melinda Gates Foundation modeled an increase in the prevalence of wasting between 10% and 50%, leading to between 250,000 and more than 1,000,000 additional child deaths.^14^ UNICEF estimates were even more dreary, stating “over 6,000 additional children under five could die each day without urgent action.”^15^ Beyond the immediate death toll, childhood malnutrition also impacts the survivors. It is well known that stunted children may never reach their full cognitive potential,^16^^,^^17^ and data from Burkina Faso show that moderate malnutrition can also impair early childhood development.^18^

As data on the continuation of nutritional health services are critical to manage the direct and indirect consequences of this ongoing pandemic, we wanted to determine the impact of the COVID-19 pandemic on the management of malnourished children in urban and rural areas of Burkina Faso.

## MATERIALS AND METHODS

We searched the handwritten registers to extract data on all children enrolled in the malnutrition program in two CRENs run by the Camillian’s Province of Burkina Faso in Ouagadougou and Nanoro between March 1, 2019 and February 28, 2022. All children younger than 5 years who had at least two weight measurements on different days were included in the analysis.

We transcribed name, sex, birthdate, and weight at enrollment in the nutrition program as well as the last date they were seen in the program and the corresponding weight in an Excel database and calculated age and total and daily weight gains. We calculated weight-for-age Z scores (WAZs) for all measurements using the WHO’s Anthro Survey Analyser (https://worldhealthorg.shinyapps.io/anthro/). We also looked at duration of treatment as a proxy for efficiency, though this parameter is often influenced by social and economic factors and comorbidities (HIV, diarrhea). Data acquisition was locked August 1, 2022.

We divided the 36 months into three 1-year periods and defined the pre-pandemic year from March 2019 to February 2020. The period from March 2020, the month when the first COVID-19 case appeared in Burkina Faso, to February 2021 was defined as the first pandemic year, and the reminder, spanning from March 2021 to February 2022, comprised the second pandemic year.

All data were entered in a unique database to allow descriptive and analytical statistics. Comparison for categorical variables was done using the χ^2^ test, and differences in continuous variables with Gaussian distribution were analyzed by the Student’s *t* test, whereas for the comparison of variables with non-Gaussian distribution, the Mann-Whitney *U* test was used. The level of significance for all statistical analysis was defined as an α of 0.05.

The primary outcome was to compare the difference in the number of patients treated in the two centers before the pandemic with the number during the first and second years of the pandemic. We also looked for differences between urban and the rural settings. The secondary outcome was any difference in the collected or calculated parameters, namely weight and Z-scores.

## RESULTS

In total, we analyzed 1,323 patient records, 731 followed at the CREN in Ouagadougou and 592 at the CREN in Nanoro (for detailed numbers, see Table 1).

**Table 1:** Comparison of demographic and anthropometric variables across the 3 study periods, separated according to urban and rural CRENs

Variable	Unit, parameter	Pre-pandemic year	*P* value (pre- vs. first year)	First pandemic year	*P* value (pre- vs. second year)	Second pandemic year	*P* value (first vs. second year)
Ouagadougou							
Patients	*n*	340	–	189	–	202	–
Age (*N* = 730)	Months, median (IQR)	12 (9, 16)	n.s.	12 (9, 16)	n.s.	12 (9, 17)	n.s.
Sex (*N* = 725)	Female/male	153/187	n.s.	87/188	n.s.	102/99	n.s.
Start weight (*N* = 731)	g, Median (IQR)	6,810 (6,115, 7,600)	n.s.	6,720 (6,090, 7,560)	n.s.	6,740 (5,950, 7,360)	n.s.
WAZ start	Median (IQR)	−2.74 (−2.15, −3.48)	n.s.	−2.81 (−2.09, −3.48)	< 0.05	−3.08 (−2.39, −3.68)	< 0.05
% Underweight	< −2 WAZ	80.0	–	78.7	–	86.7	–
Duration (*N* = 717)	Days	57 (35, 98)	< 0.001	42 (28, 75)	n.s.	63 (35, 102)	< 0.001
WAZ end	Median (IQR)	−1.36 (−0.78, −2.12)	n.s.	−1.56 (−0.98, −2.23)	n.s.	−1.58 (−0.87, −2.18)	n.s.
End weight (*N* = 718)	g, Median (IQR)	7,930 (7,300, 8,860)	< 0.05	7,750 (7,010, 8,580)	n.s.	7,980 (7,250, 8,790)	n.s.
% Underweight	< −2 WAZ	28.2	–	34.5	33.5	–	–
Total weight gain (*N* = 718)	g, Median (IQR)	1,153 (850, 1480)	< 0.0001	900 (600, 1,290)	< 0.001	1,375 (990, 1,630)	< 0.0001
Daily weight gain (*N* = 717)	g, Median (IQR)	17 (10, 28)	n.s.	19 (13, 33)	n.s.	20 (11, 33)	n.s.
Nanoro							
Patients	*n*	191	–	223	–	179	–
Age (*N* = 590)	Months, median (IQR)	12 (8, 18)	n.s.	12 (9/21)	< 0.0001	16 (11/24)	< 0.001
Sex (*N* = 573)	Female/male	76/115	n.s.	107/110	n.s.	75/90	n.s.
Start weight (*N* = 591)	g, Median (IQR)	6,710 (5,795, 7,600)	n.s.	6,500 (5,700, 7,530)	n.s.	6,600 (5,700, 7,450)	n.s.
WAZ start	Median (IQR)	−3.24 (−1.99, 4.07)	< 0.05	−3.46 (−2.65, −4.21)	< 0.0001	−3.72 (−2.98, −4.68)	< 0.01
% Underweight	< −2 WAZ	74.8	–	87.3	–	96.3	–
Duration (*N* = 589)	Days	12 (8, 18)	< 0.0001	9 (6, 14)	< 0.0001	7 (5, 10)	< 0.0001
WAZ end	Median (IQR)	−2.08 (−1.07, −3.18)	< 0.0001	−2.86 (−1.79, −3.55)	< 0.0001	−3.24 (−2.45, −4.08)	< 0.0001
End weight (*N* = 583)	g, Median (IQR)	7,500 (6,700, 8,425)	< 0.005	7030 (6,170, 8,080)	< 0.005	7,070 (6,070, 7,950)	n.s.
% Underweight	< −2 WAZ	53.4	–	71	–	87.7	–
Total weight gain (*N* = 580)	g, Median (IQR)	820 (470, 1085)	< 0.0001	490 (250, 790)	< 0.0001	435 (195, 665)	n.s.
Daily weight gain (*N* = 570)	g, Median (IQR)	57 (31, 98)	< 0.05	48 (21, 87)	n.s.	51 (24, 93)	n.s.

IQR = interquartile range; n.s. = not significant; WAZ = weight-for-age Z-score.

### Comparison of pre-pandemic year versus first year of the pandemic.

#### The HOSCO.

**Figure 1.**
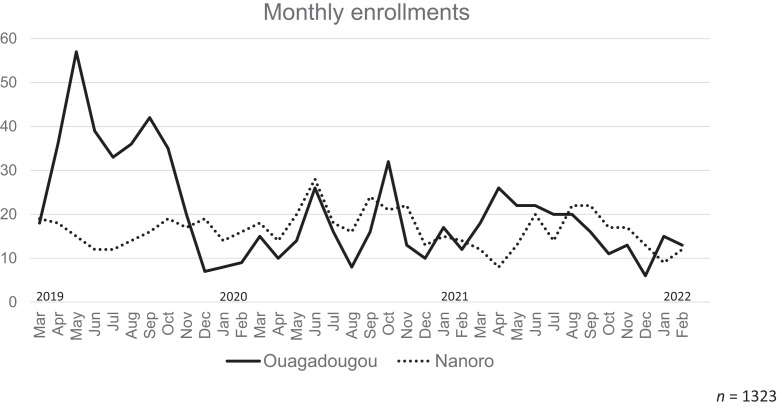
Monthly enrollments in both Centers for Rehabilitation, Education & Nutrition (CRENs) at the Hôpital Saint Camille in urban Ouagadougou and at the Center Médical Saint Camille in rural Nanoro during the 3-year study period.

In the year after the pandemic reached Burkina, the number of children seen in the CREN of HOSCO went down to almost 50%, with the lowest percentages (below 25%) in May and August 2020 (Figure 1). The monthly mean was significantly lower (*P* = 0.018).

There was no difference in sex distribution or age between the 2 years.

Comparing the weight of the children the moment they were enrolled in the nutrition program, there was only a slight difference (6,810 g in 2019 compared with 6,720 g in 2020, not significant [n.s.]). The same small difference was found in the Z-scores (−2.74 versus −2.81, n.s.).

However, Z-scores and weight at the end of treatment were lower during the pandemic (7,750 versus 7,930 g, *P* = 0.023), a significant difference that was also observed in the absolute weight gained during treatment (900 versus 1,152 g, *P* = 0.001).

The median length of treatment in Ouagadougou was 2 weeks shorter during the pandemic (42 versus 57 days, *P* = 0.001), whereas the weight gained per day of treatment was almost the same (17 versus 19 g/day, n.s.). However, both median WAZs had improved from the moderate malnutrition status (below −2) to the normal range (Z-score above −2).

#### Center Médical Saint Camille de Nanoro.

In the rural district of Nanoro, the situation was different; here, the number of children enrolled did not decrease at all. On the contrary, there was an increase of 16% over the first year (Figure 1).

Again, no differences in sex and age were noted between the two periods.

Children enrolled during the first year of the pandemic weighed less (6,500 versus 6,710 g, n.s.) and their Z-score was significantly worse (−3.24 versus −3.46, *P* = 0.011).

As seen in Ouagadougou, the weight at the end of treatment was significantly lower during the pandemic (7,030 versus 7,500 g, *P* = 0.001), as was the total weight gain (490 versus 820 g, *P* < 0.001); however, the WAZ (−2.08 versus −2.86, *P* < 0.001) indicated that most children in Nanoro did not leave the moderate malnutrition status in either period.

Here also, the duration of the treatment course was shorter during the first year of the pandemic (9 versus 12 days, *P* < 0.001), and the weight gain by treatment day decreased from 57 g to 48 g (*P* = 0.012).

### Comparison versus second year of the pandemic.

We then compared the data from the second year of the pandemic with the pre-pandemic period and found that in Ouagadougou the number of patients enrolled started to increase again (7% over the year compared with 2020) but was still significantly lower than 2019 (*P* = 0.026), reaching pre-pandemic levels by October 2021.

Regarding weight at the end of treatment, in Ouagadougou the median value returned to the pre-pandemic level and the Z-score remained stable. Duration of treatment during the second year even exceeded the pre-pandemic level, and this rebound compared with the first pandemic level was significant (*P* < 0.001). However, the weight gain per day remained stable at 19 g/day.

In Nanoro, where there had been no noticeable impact during 2020, the number declined by 7% versus 2019 and by 20% versus 2020, though neither decrease reached significance. The median age of the patients increased significantly to 16 months compared with 12 months in both precedent periods (both *P* < 0.001), but the weight at the start remained stable, indicating a more severe state of malnutrition. This is also reflected by a significant decrease in Z-score. The weight at the end of treatment remained low in the second year and thus was still significantly lower than in the pre-pandemic phase (*P* = 0.001). The picture given by the Z-scores is even worse: The WAZ at the end of treatment (−3.24) was now the same as that of the children who started treatment before the pandemic and was thus significantly lower as in both previous periods (*P* < 0.0001 for all comparisons). The total weight gained, now 435 g, was significantly lower than in 2019 (*P* < 0.001).

The deteriorating situation in Nanoro during the second year is reflected by the finding of an even shorter treatment (7 days, median), significant not only versus the pre-pandemic period but also versus the first year (both *P* < 0.001). However, the weight gain per day approached the pre-pandemic level, resting between the previous two values (n.s.).

### Comparison of urban versus rural.

**Figure 2.**
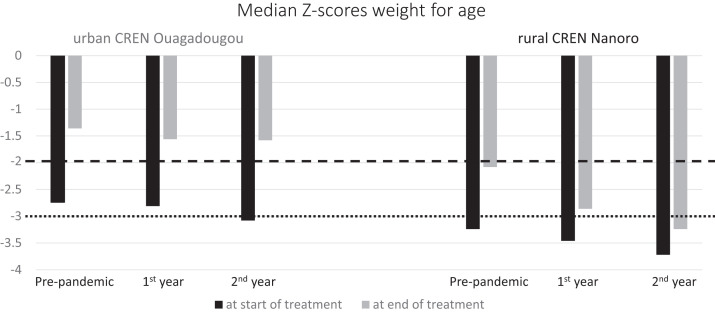
Median weight-for-age Z-scores at the start and end of treatment in the two Centers for Rehabilitation, Education & Nutrition (CRENs) before and during the first 2 years of the pandemic. The area below the dotted line = severe acute malnutrition; the area between the dashed and the dotted line = moderate acute malnutrition.

Overall, the CREN in Ouagadougou served more patients than the one in Nanoro, and although the number dropped in the first year and gained again in Ouagadougou, the situation developed inversely in Nanoro (Figure 1). Age and sex distribution did not differ significantly between the two locations in all three periods analyzed. However, there was a striking difference, consistently over the whole study period, in the length of treatment and consequently in all dependent variables: weight gain, weight at the end of treatment, and gain per day (for all comparisons *P* < 0.001). Children in the rural CREN were severely more malnourished at enrollment in the malnutrition program and had much lower Z-scores at the end of treatment. During the second year of the pandemic, their Z-scores at the end of treatment were even worse than those recorded at the start of treatment in Ouagadougou (Figure 2). The same is reflected by the percentage of children who were underweight (< −2 WZ).

## DISCUSSION

A large systematic review of the international literature has documented the huge impact on overall healthcare utilization between pandemic and pre-pandemic periods. Of all changes documented, 95.1% were a decrease, whereas only 4.9% showed an increase. The percentage change ranged from a 49% increase to an 87% decrease with a median reduction of 37.2%.^19^ This may serve as a general baseline, as no details on age groups or pathologies or world regions are presented. In fact, we also observed a sharp decline in one center as opposed to the other, where there was an increase during the first pandemic year.

The total observed reduction was in the range of what has been described on a national basis as well as in other African countries with similar age structure and childhood malnutrition problems: In Kenya, the number of underweight children attending a child welfare clinic declined by 39.6%.^20^ In Tigray, northern Ethiopia, the number of children under 5 years old screened and diagnosed with moderate or severe malnutrition went down by 28, respectively 31%.^21^ Obviously, the decrease in child consultations does not indicate a decrease in malnutrition cases: A study from Mozambique showed that although there was a 50% decline in consultations between February and October 2020, malnutrition cases increased by 100% during the same period.^22^ This was further confirmed by a telephone survey in three countries (Burkina Faso among them), where more than 20% of community participants reported having difficulty accessing preventive nutrition services or malnutrition management for their children during the COVID-19 pandemic.^23^ In that study, services provided by private hospitals were less affected by the COVID-19 pandemic. In Niamey, the capital of neighboring Niger, a study on 17 different health centers also found a reduction between 29% and 58%.^24^

COVID-19 affects nutrition not only through its direct impact on health systems but also through its impact on food systems and agriculture, starting from reductions in inputs and supplies to small-scale farming to mobility restrictions and market closures, which in turn lead to higher prices, further reducing dietary diversity and quality.^25^ A recent study from Burkina Faso (and other countries) showed that food prices increased more in rural areas than in the capital, where, in turn, assistance from the government and not-for profit organizations was higher.^26^ It is important to analyze the lessons learned from COVID-19 to be better prepared for similar events in the future.^27^

Although we did not inquire about the reasons for the significant overall decrease in patient numbers directly, we can assume that many of the fears mentioned in the introduction have come true. The difference we observed in the impact of the pandemic on the two health structures probably reflects the fact that in the capital, where a curfew and a quarantine were imposed, people were better informed and thus more afraid of being infected during a visit to the hospital. In fact, for the month of May, the same downward trend was observed in most health facilities in the capital.^28^ On the other hand, in the rural setting, where print and social media are less popular, only a few people were tested for SARS-CoV-2—and even fewer were positive, making the pandemic almost invisible on the local level. As a matter of fact, none of the districts in the central west region of Burkina Faso, where Nanoro is located, reported a decrease in attendance at health facilities due to COVID-19.^25^ This finding is contrary to a huge multicountry assessment (including Burkina Faso) on health service utilization reported in the routine national health information systems, where rural areas seemed to be more affected than the capital cities; however, they found a very low impact on the utilization of maternal and child health preventive interventions (−2% to −6%) and did not report on the management of malnutrition.^29^

Interestingly, although fewer children visited the HOSCO during the pandemic, they were not more severely malnourished. This indicates that there was no selection of more severe cases, as opposed to what was expected. The opposite is true for Nanoro.

The duration of the treatment was shorter, and consequently weight gain was less during the pandemic in both settings, probably because patients were discharged to the community earlier by the staff to avoid crowding in the CREN. Particularly in Ouagadougou, where the Z-score at the end of treatment did not differ significantly, with almost all children passing from moderate malnutrition to the normal range (a WAZ of −2 or better) in all three periods, the shorter duration probably was accompanied by greater efficiency. In contrast, in Nanoro, where the children started treatment with a severely worse Z-score before the pandemic, most were discharged while being still moderately malnourished. Patients arriving at the rural CREN in the second year of the pandemic were more seriously malnourished than those followed previously, indicating that the lockdown possibly affected available nutrition there. Furthermore, in Nanoro, Z-scores at the end of treatment continued declining, showing that duration of treatment there was in fact too short. The consistently shorter treatment in the rural environment may have several explanations, among them fewer means for transportation, less available time for the caregiver to spend in the CREN because of siblings and agricultural fieldwork, and/or a different perception of a healthy child’s body image. It should be considered that some caregivers did not complete treatment in the CREN once the children had started to improve.

In the second year, information on the pandemic reached the rural areas and was now perceived as a greater threat, whereas in the capital (as in many European cities), people somehow had started to cope with the situation and had received more assistance. Vaccines probably also contributed to that difference, because they first arrived in Ouagadougou (and the district capitals) by the end of May 2021 and afterward were distributed to the community health centers to make them easily accessible in the rural areas of the country.

Our study has several limitations, mainly the fact that data on comorbidities and long-term follow-up data, including the final outcome of the children (survival), have not been registered in the original database and thus are not available for analysis. However, this is the first head-to-head comparison of a rural and an urban CREN treating malnourished children by the same standard and with similar means. Thus, we are able to clearly document the important existing difference between urban and rural settings in terms of the impact of COVID-19 on undernourished children in the Sahel.

Our data help to fill the gap identified in a recent scoping review by Adu et al.,^30^ who found limited research on how the COVID-19 pandemic affected maternal and child health in relation to healthcare access and utilization in Africa.

Finally, we want to underline that even during the pandemic, childhood undernutrition remained treatable and that every action must be taken^31^ to prevent this condition from the beginning as an important step in growing resilient and healthy children, which will allow populations to better resist this and future pandemics.
